# A rapid and versatile tool for genomic engineering in *Lactococcus lactis*

**DOI:** 10.1186/s12934-019-1075-3

**Published:** 2019-01-31

**Authors:** Tingting Guo, Yongping Xin, Yi Zhang, Xinyi Gu, Jian Kong

**Affiliations:** State Key Laboratory of Microbial Technology, Shandong University, No. 72 Binhai Road, Qingdao, 266237 People’s Republic of China

**Keywords:** *Lactococcus lactis*, ssDNA recombineering, CRISPR/Cas9 counterselection, Genomic engineering

## Abstract

**Background:**

*Lactococcus lactis* is one of the most extensively characterized lactic acid bacteria, from physiological traits to industrial exploitation. Since last decade, *L*. *lactis* has been developed into cell factories for the production of bioactive compounds such as enzymes, vaccine antigens and natural products. However, its precise and efficient genome editing tools is still required to make *L*. *lactis* more suitable candidate for engineered functionality.

**Results:**

A high active recombinase, RecT of *Enterococcus faecalis* ATCC14506, was selected from six candidates and mediated homologous recombination between single-stranded DNA (ssDNA) and the *L*. *lactis* chromosomal *rpoB* locus with an efficiency of 100% after rifampin selection. To screen mutants without an externally selectable phenotype, the CRISPR/Cas9 system was used for counterselection, yielding an *upp* mutant with an efficiency of 46%. By optimization of the copy number of plasmid carrying the CRISPR/Cas9 system and the length of spacer sequence, the off-target efficiency of the *recA*, *galK*, *hemN* and *noxD* genes were eliminated. The ability of this optimized tool to perform sequential point mutation was demonstrated using the *upp* and *galK* gene loci as targets with improved efficiencies > 75%. Moreover, seamless genomic DNA deletions (50/100 bp) or insertion (a *loxP* site, 34 bp) was efficiently accomplished within 72 h.

**Conclusions:**

The work provided a rapid, versatile and precise tool for *L*. *lactis* genomic engineering by combination of ssDNA recombineering with improved CRISPR/Cas9 counterselection. This tool will simplify the production of isogenic strains for assessment of gene function or construction of biosynthetic host.

**Electronic supplementary material:**

The online version of this article (10.1186/s12934-019-1075-3) contains supplementary material, which is available to authorized users.

## Background

Lactic acid bacteria (LAB) are a group of non-sporulating Gram-positive bacterium that catabolize water soluble carbohydrates mainly into lactic acid, including *Lactococcus*, *Lactobacillus*, *Streptococcus*, *Enterococcus*, *Pediococcus*, *Leuconostoc* and *Oenococcus*. They are highly diverse in genetic composition and physiological metabolism [1]. Therefore, the developed genome editing tools in LAB usually performed in a strain specific manner [2–5].

*Lactococcus lactis* is one of the most commonly used strain in the manufacture of fermented dairy products [6]. More importantly, *L*. *lactis* is an ideal cell factory for the production of recombinant proteins and natural products because of its GRAS (generally regarded as safe) status, relatively small genome and simple metabolism [7]. As with deciphering of unknown genes and uncovering of novel biosynthetic pathway, it is key that *L*. *lactis* genome can be efficiently edited to fine modify a specific gene or reroute a natural metabolic pathway for desirable end products. Thus, genomic modification is indispensable, including precise point mutation, deletion and insertion of the target genes. Unfortunately, precise genomic modification of *L*. *lactis* is more difficult compared with other LAB strains, such as *Lb*. *reuteri*, *Lb*. *casei* and *Lb*. *plantarum* [2–5].

Nowadays, the common techniques for editing *L*. *lactis* genome is mainly to rely on RecA-dependent homologous double-crossover events with nonreplicative or conditional replicative plasmids [8]. Although practicable, the RecA-dependent recombination occurs rare, leading to long subcultivation and laborious screening for final mutants [9]. Several modified approaches have been developed to increase the screening efficiency by counterselectable markers, such as the genes *upp*, *oroP*, and *pheS* [10–12]. Nevertheless, these protocols are still imperative performed in two steps (vector integration and co-integrate resolution) and labor-intensive, time-consuming (usually 3 weeks) to generate a prospective mutant.

Prophage-derived recombinase operons have been exploited to improve the efficiency of genome editing in several bacteria [13–16]. The well-characterized λ-Red system, consisting of Redα (5′–3′ exonuclease), Redβ (single-stranded DNA binding protein) and Redγ (host nuclease inhibitor), has been used for genomic engineering in *E*. *coli* [14]. Recently, two λ-Red like operons were explored from *Lactobacillus* prophages, and applied for construction of the genome editing systems [4, 5]. Redβ or its analogs RecT can mediate recombination through a ssDNA oligonucleotide which preferentially binds to the lagging strand during DNA replication (ssDNA recombineering), generating subtle genomic mutations [17, 18]. To our knowledge, a functional λ-Red like system has not been reported in *L*. *lactis*, while ssDNA recombineering shows great potential to engineer its genome [19]. Considering the diversity and host specificity of the ssDNA binding protein [20], termed recombinase, seeking functional RecT proteins will greatly improve the homologous recombination efficiency and accelerate the development of the genetic manipulation platform in *L*. *lactis*. At the same time, an efficient selection system is still needed for easily screening the expected recombinant.

Clustered regularly interspaced short palindromic repeat (CRISPR)/Cas9 system has been used as a mutagenesis screening strategy in *L*. *lactis* [21]. This system simply requires Cas9, tracrRNA and crRNA carrying a 20–30 bp spacer target to the chromosomal site [22]. Cas9 is guided to search the protospacer-adjacent motif (PAM), binds and then induces double-stranded break (DSB) at the target site [23]. When used as a counterselectable marker, Cas9-induced DSB at the wild type allele provides fast screening of expected mutant [21]. However, some wild type cells might escape from the lethality of Cas9, probably leading to the false positive subpopulation. And, it is still difficult to obtain a point mutation, deletion or insertion mutant by CRISPR/Cas9 counterselection because of the low efficiency of the endogenous homologous recombination in *L*. *lactis*. To obtain the final mutation efficiently and simply, the CRISPR/Cas9-assisted ssDNA recombineering system has been reported in *Lb. reuteri* [3], while it is still not applied to *L. lactis*.

In this work, according to the previous reports and available prophage sequence information on NCBI database [4, 5, 14, 17–19, 24], the recombination activities of six known and predicted recombinases were tested in *L*. *lactis*. And, the specificity and efficiency of Cas9-induced DSB was improved. By combining the ssDNA recombineering with CRISPR/Cas9 counterselection, a tool was developed to meet the urgent requirement for an efficient and reliable system for *L*. *lactis* precise genomic engineering.

## Results

### Selection of recombinase suitable for ssDNA recombineering in *L*. *lactis*

**Fig. 1:**
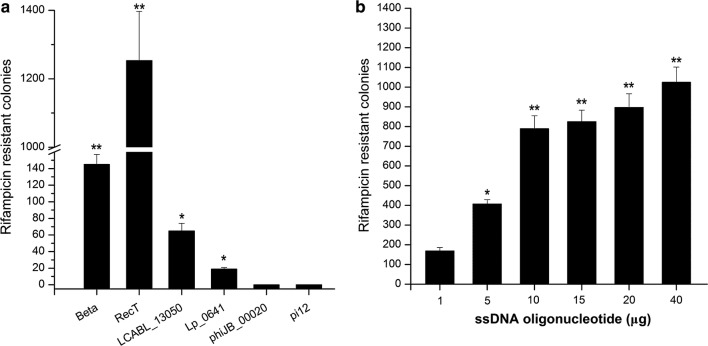
SsDNA recombineering in *L*. *lactis*. **a** Activity of six recombinases when expressed exogenously in *L*. *lactis*. Recombineering utilized synthetic ssDNA introducing rifampin resistance. **b** Effects of various ssDNA dosages on recombination efficiency. Recombination frequencies significantly higher are indicated by an asterisk (*P < 0.05; **P < 0.01). The values are means ± standard deviations of three independent experiments

The activities of six characterized or predicated prophage-derived recombinases were tested by evaluating their function to mediate ssDNA recombineering of the *rpoB* H486N mutation conferring cells with rifampin resistance [19]. In *L*. *lactis* NZ9000, the nisin of 10 ng/mL was used to induce the expression of the six candidates: Redβ, recombinase of *E*. *coli* λ-Red system; RecT, derived from *E*. *faecalis* ATCC14506; LCABL_13050 and Lp_0641, recombinases from *Lb*. *casei* BL23 and *Lb*. *plantarum* WCFS1, respectively; phiJB_00020, a predicated ssDNA binding protein derived from *Lactobacillus* prophage; pi12, a putative recombinase identified on the basis of protein homology from *L*. *lactis* IL1403 [4, 5, 25–27]. After electroporation of 100 μg ssDNA oligonucleotide rpoBo1 into *L*. *lactis* NZ9000 cells expressing the recombinase, the rifampicin-resistance colonies were counted. As shown in Fig. 1a, a total of 145, 1253, 65, 19, 0 and 0 colonies were obtained in three independent experiments mediated by Redβ, RecT, LCABL_13050, Lp_0641, phiJB_00020 and pi12, respectively. The RecT showed the highest recombineering activity, and 20 randomly picked colonies were confirmed to carry desired mutations by MAMA-PCR and sequencing analysis (Additional file 1: Fig. S1). Moreover, decrease of the ssDNA oligonucleotide dosage showed that as low as 10 μg generated almost the similar number of recombinants compared with 100 μg (Fig. 1b). Therefore, the RecT was selected for ssDNA recombineering in *L*. *lactis*.

### CRISPR/Cas9 as counterselection marker after ssDNA recombineering

**Fig. 2:**
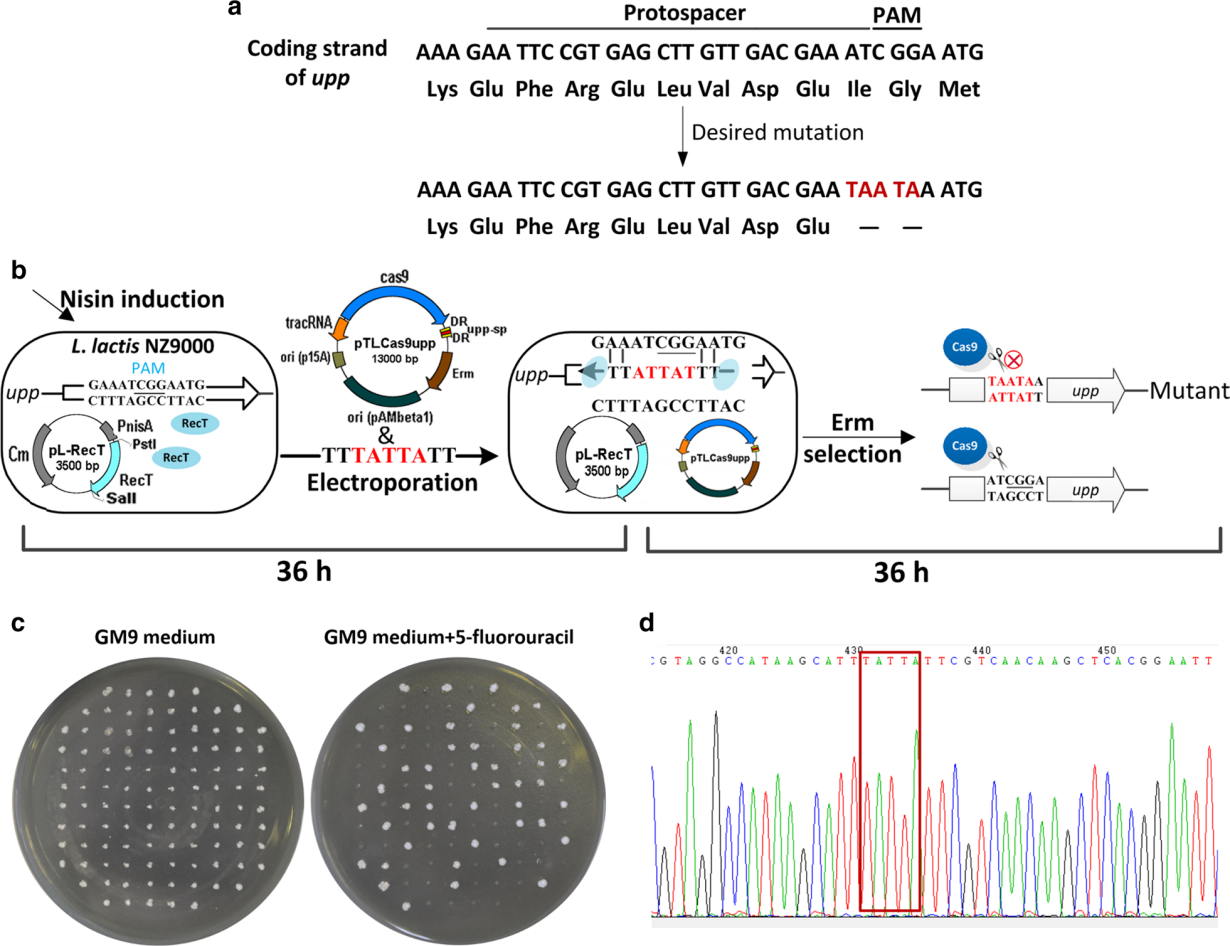
CRISPR/Cas9 as counterselection marker after ssDNA recombineering. **a** Sequence of the targeted *upp* region of *L*. *lactis* NZ9000 is shown aligned with amino acids specified by each codon. If the desired mutation (red bases) is introduced, two successive stop codons will be produced. **b** Overview of ssDNA recombineering coupled CRISPR–Cas9 counterselection. The bases of PAM are underlined. In the first 36 h, competent *L*. *lactis* cells expressing recombinase RecT was prepared, and transformed with ssDNA uppo and pTHCas9upp simultaneously by electroporation. After transformation, the cells were recovered in GM17 agar containing erythromycin for 24 h. In the second 36 h, the colonies were picked up, and the expected mutation was PCR amplification and subjected to sequencing analysis. **c** Re-screening of the recombineered colonies using 5-fluorouracil. The Δ*upp* mutants containing desired mutations were capable of growing on GM9 agar containing 10 μg/mL 5-fluorouracil, but the WT was unable to grow. GM9 agar was used as a control. **d** Sequencing of the Δ*upp* mutant. The bases in the red box are mutations

To achieve recovery of mutants without an antibiotic resistant phenotype, the CRISPR/Cas9 system from *Streptococcus pyogenes* was applied to kill wild type cells after ssDNA recombineering. As previously reported [28], three genetic elements Cas9, tracrRNA and crRNA were introduced into a low copy number plasmid pTRKL_2_ (Erm^r^), generating pTLCas9. Recombinant cells harboring the desired mutation on the spacer sequence or the PAM site can survive in the presence of erythromycin. The first trial aimed to bring five successive bases (from 106 nt to 110 nt) mutation in the uracil phosphoribosyltransferase (UPRTase) gene *upp*, which would generate two proximate termination codons (Fig. 2a), conferring cells with 5-fluorouracil resistance. Following the nisin induced expression of RecT in *L*. *lactis*, the ssDNA uppo and the targeting plasmid pTLCas9upp were co-electroporated (Fig. 2b), generating 106 erythromycin resistant colonies per 10^9^ competent cells. A control was set by co-electroporation of the ssDNA uppo and pTLCas9 containing an invalid spacer, yielding 1.44 × 10^5^ erythromycin resistant colonies per 10^9^ competent cells. Then, the colonies of the experiment group were all picked and re-screened by 5-fluorouracil. As a result, 49 of them were survive in the presence of 5-fluorouracil and contained desired mutations by sequencing confirmation (Fig. 2c, d), while none of the randomly picked colonies of the control group was alive (data not shown). These results demonstrated the ssDNA recombineering coupled CRISPR/Cas9 counterselection could achieve genomic point mutations. Meanwhile, the final mutant efficiency was 46%, indicating an off-target effect of CRISPR/Cas9 in *L*. *lactis*.

### Reduction of the off-target effects of CRISPR/Cas9 in *L*. *lactis*

**Fig. 3:**
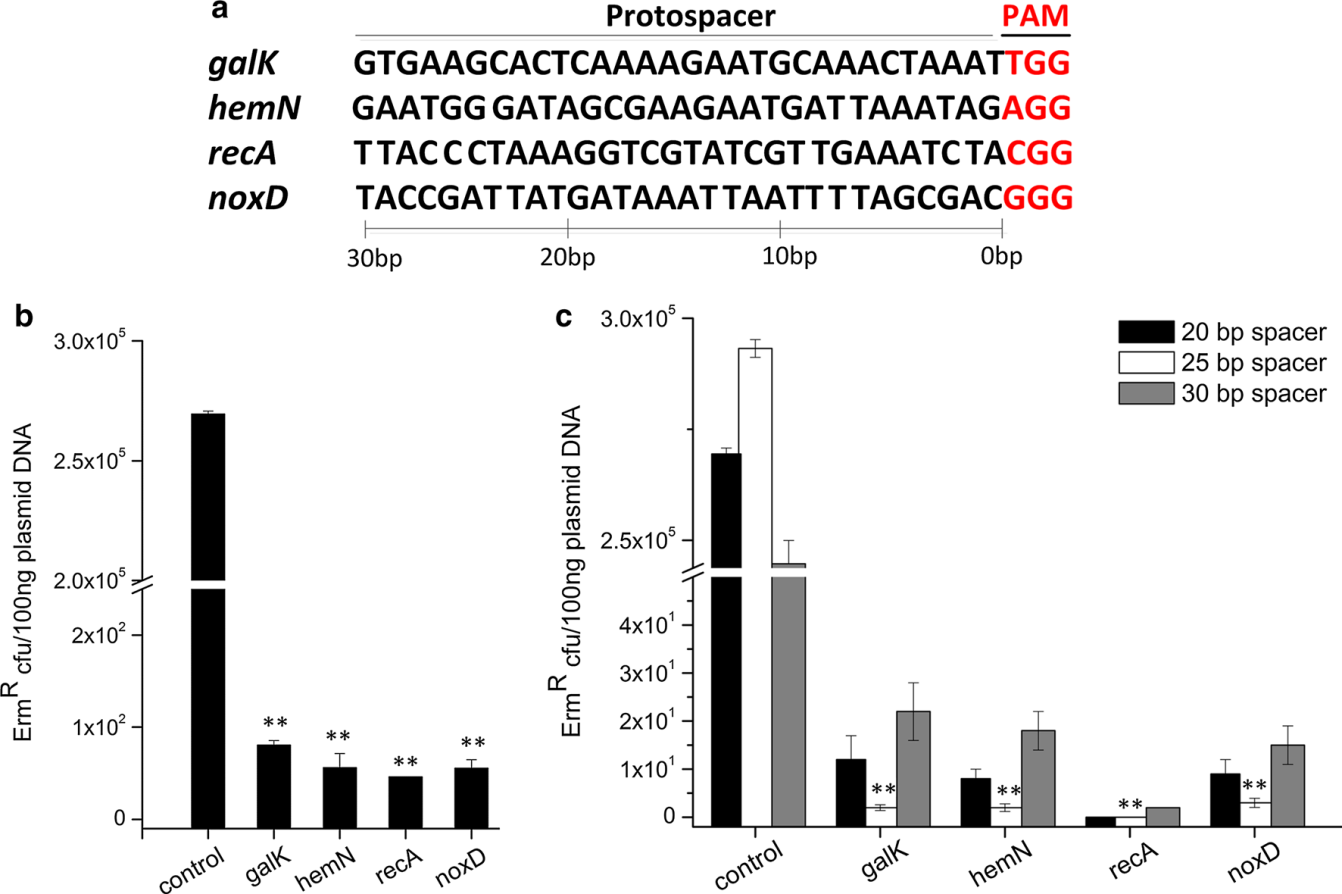
Application and improvement of CRISPR/Cas9 system in *L*. *lactis* NZ9000. **a** The nucleotide sequences of protospacers and PAM sites in the four genomic locations to be targeted. **b** The targeting efficiency by the low copy number vector pTRKL_2_ based CRISPR/Cas9 system. When transforming NZ9000 with pTLCas9galk1 (or pTLCas9hemN1, pTLCas9recA1, pTLCas9noxD1), there was a 10^5^-fold reduction in viable colonies compared to transformation with pTLCas9 (control). **c** Reduction of the off-target effects in *L*. *lactis*. The Cas9, tracrRNA and crRNA were carried by the high copy number plasmid pTRKH_2_. The spacer sequence was set as 20 bp, 25 bp and 30 bp for each of the four genomic locations. Statistically significant differences are indicated by asterisks (*P < 0.05; **P < 0.01). The values are means ± standard deviations of three independent experiments

The targeting efficiency of pTRKL_2_ based CRISPR/Cas9 system was firstly analyzed at four individual genomic locations included in the *galK*, *hemN*, *recA* and *noxD* genes of *L*. *lactis* NZ9000 (Fig. 3a). Electroporation of the control plasmid pTLCas9 generated the transformants of 2.72 × 10^5^ cfu/100 ng plasmid DNA, while 80.5, 56.0, 46.0 and 55.5 transformants were yielded with the plasmids containing valid spacers of the four locations, respectively (Fig. 3b). Then, 12 survived colonies from each location were selected and confirmed that there was no mutation in the spacer and PAM by sequencing analysis (data not shown). These results confirmed that the pTRKL_2_ based CRISPR/Cas9 system had the DSB activity in *L*. *lactis*, while the off-target effects remain, which would result in false positive subpopulation following recombineering process.

Then, a high copy number plasmid pTRKH_2_ was used to increase the concentration of the CRISPR/Cas9 elements. As shown in Fig. 3c, the targeting efficiency of the *recA* gene increased to 100%, and the efficiencies of the *galK*, *hemN* and *noxD* genes were also enhanced by sevenfold. Furthermore, the length of spacer was also optimized. Extending the spacer from 20 to 25 bp basically eliminated the off-target phenomenon for the *galK*, *hemN* and *noxD* genes, while further extension unexpectedly increased the off-target effects.

To test the efficiency of the improved CRISPR/Cas9, the designed *upp* mutation was performed again, generating 58 erythromycin resistant colonies. 50 out of 58 colonies were resistant to 5-fluorouracil and contained desired mutations by sequencing confirmation, yielding a final mutation efficiency of 87% (data not shown). Therefore, a new genomic engineering tool was developed by combination of RecT-mediated ssDNA recombineering with the efficient CRISPR/Cas9 counterselection in *L*. *lactis*.

### *Lactococcus lactis* genomic engineering by ssDNA recombineering coupled the efficient CRISPR/Cas9 counterselection

#### Sequential precise point mutation in *L*. *lactis*

**Fig. 4:**
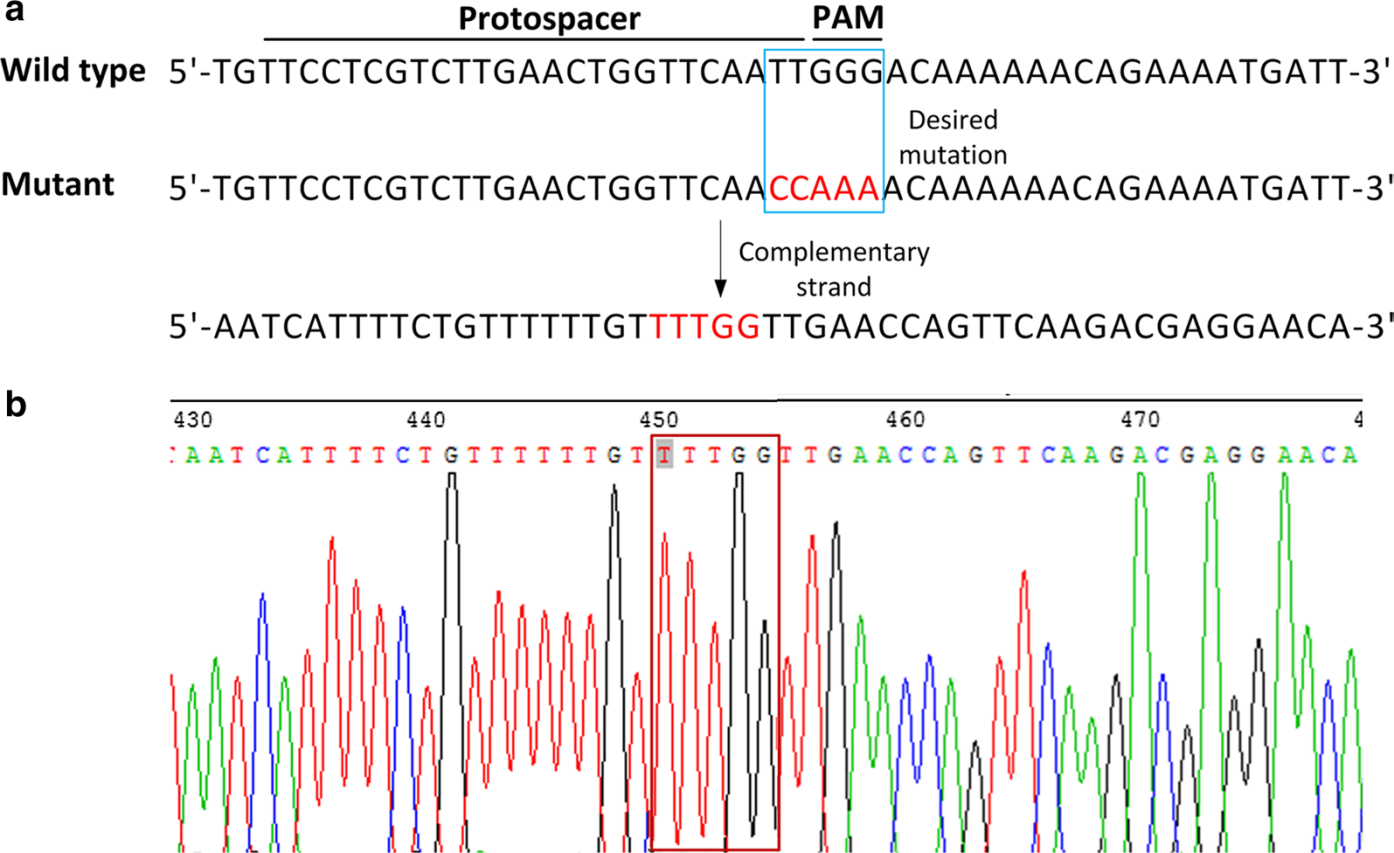
Sequential precise point mutation of the *galK* gene in *L*. *lactis* with the Δ*upp* mutations. **a** Schematic diagram of site mutagenesis of the *galK* gene in the Δ*upp* mutant. **b** Sequencing analysis of the colonies randomly selected by CRISPR/Cas9 counterselection

To perform precise point mutation of the *galK* gene in the Δ*upp* mutant strain, the targeting plasmid pTHCas9upp used for the *upp* mutation selection was firstly cured. Subsequently, an ssDNA oligonucleotide galKo targeting the *galK* gene was designed, which would bring in the bases mutation of the selected PAM sequences (Fig. 4a). After co-electroporation of the ssDNA galKo and the targeting plasmid pTHCas9galK into the Δ*upp* mutant cells expressing RecT, the culture was recovered in the medium with erythromycin. 12 colonies were picked, and 9 of them contained the designed mutation by sequencing confirmation (Fig. 4b). This result confirmed the tool developed here permits iteratively genomic point mutation.

#### Short chromosomal DNA deletion

**Fig. 5:**
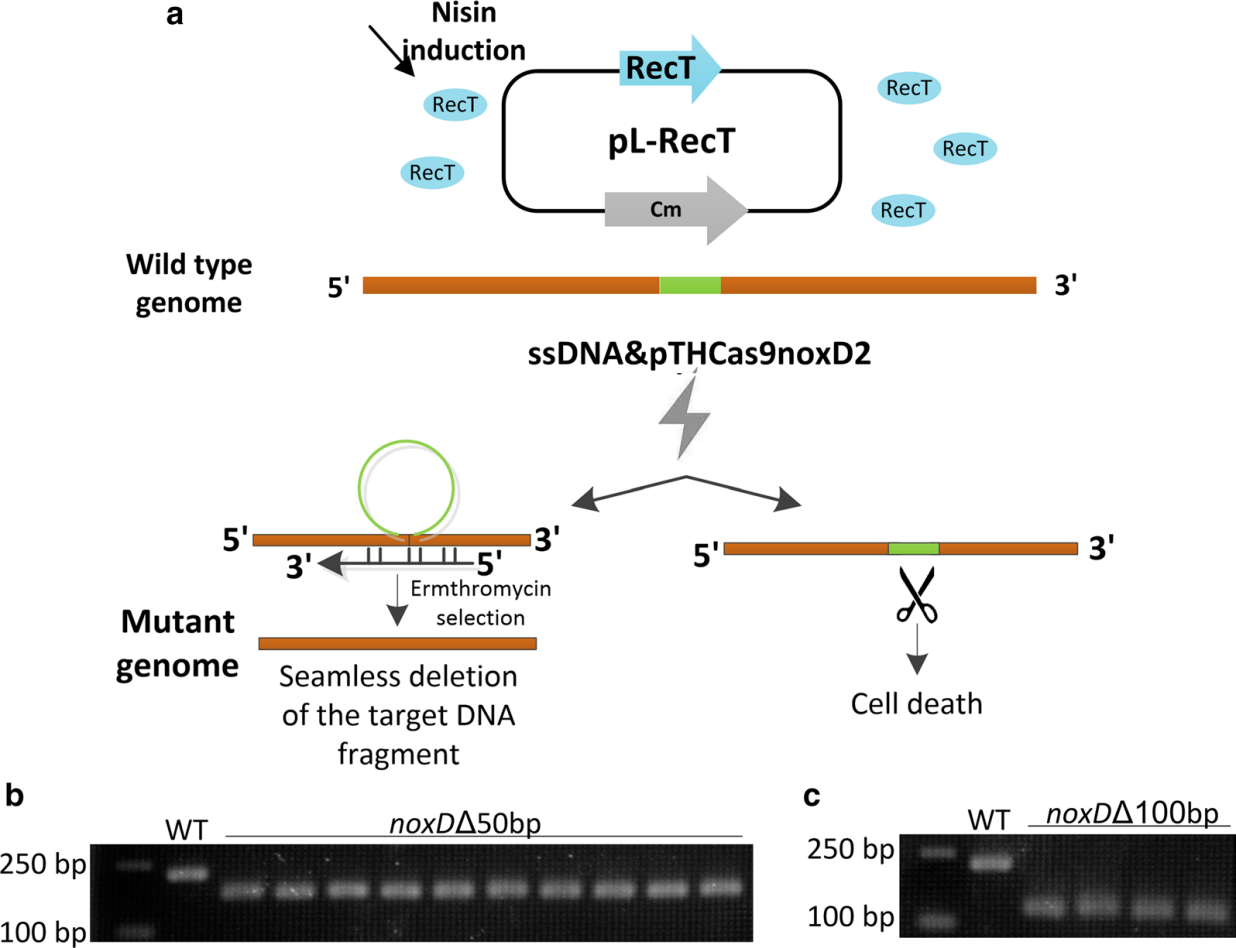
Short DNA fragment deletion from the *L*. *lactis* genome. **a** Schematic diagram of deletion of partial *noxD* gene in *L*. *lactis* NZ9000. **b**, **c** PCR amplification of the *noxD* locus from randomly selected colonies surviving under erythromycin selection. The wild type corresponds to an amplicon 240 bp in size, whereas the 50 bp and 100 bp in-frame deletion result in products at 190 bp (**b**) and 140 bp (**c**) in size. DL 5,000 ladder (Takara, Japan) is shown

To carry out chromosomal DNA deletion in a seamless manner, the pseudogenes *noxD* was selected as the target because the gene was non-essential and its deletion would not negatively affect cell growth [29]. An 80 bp disruption oligonucleotide was designed with 40 bp homology arms upstream and downstream flanking of the deletion region which contained targeting sequence of plasmid pTHCas9noxD2 (Fig. 5a). Co-electroporation of oligonucleotide noxDo1 and pTHCas9noxD2 into the NZ9000 cells expressing RecT yielded 16 colonies per 10^9^ cells. 10 colonies were picked and demonstrated to be the desired mutant with 50 bp in-frame deletion in the *noxD* (Fig. 5b). Furthermore, the deletion region was extended to 100 bp, generating 4 colonies (all expected) per 10^9^ cells (Fig. 5c). These results confirmed that this tool could be applied to perform short genomic DNA fragment deletion seamlessly within 72 h.

#### Short DNA insertion

**Fig. 6:**
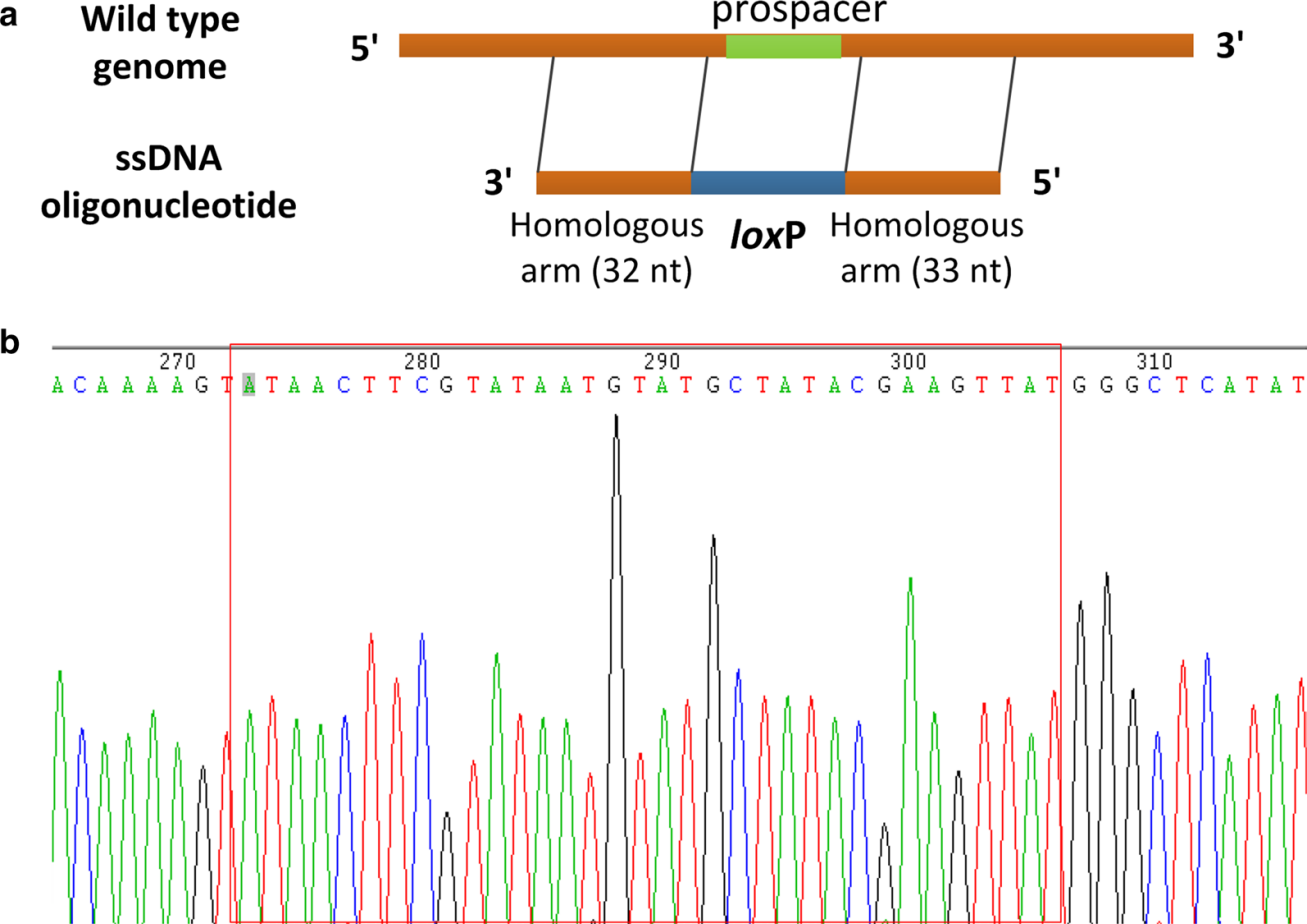
Short DNA insertion into the *L*. *lactis* genome. **a** Schematic diagram of the *loxP* site insertion into the *noxD* gene. **b** Sequencing confirmation of *loxP* insertion

To test the feasibility of this tool to insert short DNA fragment into the genome of *L*. *lactis* NZ9000, the 34 bp *loxP* site was selected as a target. A 99 bp oligonucleotide was designed with 34 bp *loxP* sequence clamped by 33 bp and 32 bp homologous arms upstream and downstream of the insertional location (Fig. 6a). After co-electroporation of the ssDNA noxD-loxPo and the targeting plasmid pTHCas9noxD2 into the NZ9000 cells expressing RecT, two erythromycin resistant colonies were obtained. As shown in Fig. 6b, sequencing analysis of the two colonies showed that the *loxP* site was correctly inserted into the genome of colony 1, while the insertional sequence in the genome of colony 2 could partly match the *loxP* site. These results indicated the developed tool could accomplish insertion of short DNA sequence into the genome of *L*. *lactis*.

## Discussion

*Lactococcus lactis* is an important model for LAB genetics, physiological, systematic, and applicable research, while its efficient, rapid and precise genomic modification remains yet to be addressed. In this work, we described a tool for genome engineering which incorporates the ssDNA recombineering and CRISPR/Cas9 targeting, reported for the first time in *L*. *lactis*. Through selecting suitable recombinase and reducing the off-target effect of Cas9-induced DSB, the success rate of genomic mutation was significantly improved to > 75% in *L*. *lactis* within 72 h. In contrast to the existing approaches, this protocol is highly efficient, time-saving and easy-to-use for introducing precise point mutations and performing gene deletion and insertion in a seamless manner.

The primary limitation of the established genomic modification tool of *L*. *lactis* is the low efficiency of RecA-dependent homologous recombination [30]. It is inevitable to find exogenous recombination systems. Nowadays, two kinds of prophage-derived systems have been demonstrated their recombinational function in LAB, including λ-Red like operons (using dsDNA substrate) and ssDNA recombinase (using ssDNA substrate) [4, 5, 19, 20]. Here, we were not able to obtain the recombination activity of a putative λ-Red like operon (pi10-11-12) from *L*. *lactis* IL1403 (data not shown), neither the predicted ssDNA recombinase encoded by pi12 in *L*. *lactis* NZ9000. Our failure to identify a *L*. *lactis* recombinase suggested that recombinases native to *L*. *lactis* might be markedly dissimilar in sequence and structure to those which had the activity for ssDNA recombineering [31]. Fortunately, the four recombinases characterized in other organisms were all workable, of which the RecT from *E*. *faecalis* ATCC14506, sharing 42.81% identity to the RecT from *E*. *faecalis* V583 [24], showed the highest recombination activity in *L*. *lactis* NZ9000. These results agreed with the previous opinion that RecT from *E*. *faecalis* was efficient in phylogenetically distant bacteria and was a highly efficient recombinase [20]. Moreover, the less ssDNA (as few as 10 μg) and shorter homologous arm (as short as 32 bp) were required for the selected RecT to mediate ssDNA recombineering compared with the previous works [19, 20], which could reduce the synthetic cost of ssDNA substrate.

CRISPR/Cas is an adaptive immune system in bacteria and archaea against foreign genetic elements [32]. The type II CRISPR/Cas9 system from *S*. *pyogenes* has been exploited as a facile and programmable platform for genomic manipulation in a sequence-specific manner in eukaryotes and some prokaryotes [3, 23, 33, 34]. The off-target effect is a common challenge during application of the CRISPR–Cas9 system [35]. Previous reports demonstrated that several factors may influence the targeting efficiency, including the type of host cell, the composition of spacer sequence and the activity of Cas9 [35–37]. To our knowledge, the function of CRISPR/Cas9 has been demonstrated in *L*. *lactis*, *Lb*. *casei*, *Lb*. *reuteri* and *S*. *thermophilus*, while the off-target effect of Cas9 has not been discussed and yet to be addressed [3, 6, 21, 38]. Here, we proved that the specificity and efficiency of Cas9-induced DSB in *L*. *lactis* genome is tightly related to the concentration of CRISPR/Cas9 system and the length of spacer sequences. Through an appropriate increase in the intercellular concentration of CRISPR/Cas9 system and optimization of the length of spacer could yield a more efficient CRISPR/Cas9 based counterselection system compared with that in the previous report [21].

Based on the RecT-mediated ssDNA recombineering coupled with the efficient CRISPR/Cas9 counterselection, a new genomic engineering tool was proposed for *L*. *lactis*. The workflow of this tool is simple, handling and can be accomplished within 72 h. Moreover, as the plasmid carrying the CRISPR/Cas9 elements was easily cured, sequential engineering was allowed. This facility provided prerequisite for high-throughput genome engineering in *L*. *lactis*. At the same time, this work revealed several interesting challenge of *L*. *lactis* genomic modification. First, a targeting plasmid containing two spacers with *upp* and *galK* was constructed, and the possibility to simultaneously edit multiple sites in the genome which was feasible in *E*. *coli* was also tested [39], but failed in *L*. *lactis* (data not shown). We speculated that the failure might be due to the limitations of the homologous recombination of the RecT. Future improvements of the homologous recombination system in *L. lactis* may allow simultaneous multi-locus editing of the genome. Second, compared with point mutation, gene deletion mediated by the tool developed here was less efficient by about 100-fold in *L*. *lactis*, and the deletion of longer chromosomal DNA (150 bp) did not success. These phenomena were also observed in other organisms [19]. We speculated that the binding capacity of oligonucleotide for gene deletion might be weaker than that for point mutation, leading to relative low recombination efficiency. Third, one of the two colonies produced during *loxP* site insertion could partly matched the *loxP* site, suggesting that unexpected mutation could be brought in the synthetic of long ssDNA oligonucleotide (99 bp). Fourth, due to restrictions in the length of synthetic ssDNA substrate, large DNA fragment cannot be inserted into the chromosome through the method developed here. Fortunately, the engineered *L*. *lactis* with an intact *loxP* site constructed here will be applicable for gene insertion mediated by the Cre/*loxP* site-specific recombination system [40].

## Conclusions

In summary, a new tool was developed to mediate synthetic oligonucleotide into the *L*. *lactis* genome by ssDNA recombineering and kill cells without expected mutations by CRISPR/Cas9 counterselection. Through selecting a suitable recombinase and improving the CRISPR/Cas9 lethality, this tool can achieve precise point mutation, seamless deletion and insertion at efficiencies of > 75% within 72 h. This powerful tool will fulfill high-throughput genetic engineering and facilitate studies to peek deep into the role of specific genes and reveal their novel function in *L*. *lactis*.

## Methods

### Bacterial strains and growth conditions

Bacterial strains and plasmids used in this study are listed in Table 1. *L*. *lactis* strains NZ9000, its mutants and IL1403 were routinely grown in M17 medium (Oxoid, Basingstoke, United Kingdom) supplemented with 0.5% (wt/vol) glucose (GM17) at 30 °C under static conditions. When needed, *L*. *lactis* was grown in semi-defined M9 medium supplemented with 0.4% glucose (GM9) at 30 °C under static conditions [41]. *Enterococcus faecalis* ATCC14506, *Lb*. *casei* BL23, *Lb*. *plantarum* WCFS1*, Lb*. *delbrueckii* SDMCC050201 were grown statically at 37 °C in MRS medium (Oxoid, Basingstoke, United Kingdom). *Escherichia coli* strains were grown aerobically at 37 °C in Luria–Bertani (LB) medium. If necessary, chloramphenicol (Sangon, China) was used at 5 μg/mL for *L*. *lactis* or 10 μg/mL for *E*. *coli*, erythromycin (Sangon, China) 10 μg/mL for *L*. *lactis* or 250 μg/mL for *E*. *coli*, rifampicin (Sangon, China) 25 μg/mL for *L*. *lactis*. Nisin (Sigma, USA) was added to a final concentration of 10 ng/mL. 5-fluorouracil (Sigma, USA) was added to a final concentration of 10 μg/mL.

**Table 1:** Bacterial strains and plasmids used in this study

Strain or plasmid	Relevant features	Source or references
Strains		
*E*. *coli* DH5α	Subcloning host	Our lab
*E*. *coli* XL1-Blue	Subcloning host	Our lab
*L*. *lactis* NZ9000	*L. lactis* MG1363 pepN::nisRK; commonly used host for NICE system	[43]
*L*. *lactis* IL1403	The source of putative recombinase pi12	[27]
*E*. *faecalis* ATCC14506	The source of recombinase RecT	Our lab
*Lb*. *plantarum* WCFS1	The source of recombinase Lp_0641	[4]
*Lb*. *casei* BL23	The source of recombinase LCABL_13050	[5]
*Lb*. *delbrueckii* SDMCC050201	The source of putative recombinase phiJB_00020	[26]
Plasmids		
pLeiss:Sec:Nuc	*L. lactis* and *E*. *coli* shuttle cloning vector which contains PnisA promoter used for nisin controlled gene expression; Cm^r^	[44]
pTRKL_2_	*L. lactis* and *E*. *coli* shuttle cloning vector which exists at low copy number in *L*. *lactis*; Erm^r^	[45]
pTRKH_2_	*L. lactis* and *E*. *coli* shuttle cloning vector which exists at high copy number in *L*. *lactis*; Erm^r^	[45]
pCas9	Low copy number vector, expression of Cas9 nuclease, tracrRNA and crRNA in *E*. *coli*; Cm^r^	[23]
pKD46	λ Red recombinase expression vector; the source of recombinase Beta; Amp^r^	[46]
pTLCas9	Cas9, tracrRNA and crRNA cloning in pTRKL_2_; Erm^r^	This work
pTHCas9	Cas9, tracrRNA and crRNA cloning in pTRKH_2_; Erm^r^	This work
pTLCas9upp	pTLCas9 with a 20 bp spacer targeting the *upp* gene of *L*. *lactis* NZ9000; Erm^r^	This work
pTLCas9galk1	pTLCas9 with a 20 bp spacer targeting the *galK* gene of *L*. *lactis* NZ9000; Erm^r^	This work
pTLCas9hemN1	pTLCas9 with a 20 bp spacer targeting the *hemN* gene of *L*. *lactis* NZ9000; Erm^r^	This work
pTLCas9recA1	pTLCas9 with a 20 bp spacer targeting the *recA* gene of *L*. *lactis* NZ9000; Erm^r^	This work
pTLCas9noxD1	pTLCas9 with a 20 bp spacer targeting the *noxD* gene of *L*. *lactis* NZ9000; Erm^r^	This work
pTHCas9galk1	pTHCas9 with a 20 bp spacer targeting the *galK* gene of *L*. *lactis* NZ9000; Erm^r^	This work
pTHCas9hemN1	pTHCas9 with a 20 bp spacer targeting the *hemN* gene of *L*. *lactis* NZ9000; Erm^r^	This work
pTHCas9recA1	pTHCas9 with a 20 bp spacer targeting the *recA* gene of *L*. *lactis* NZ9000; Erm^r^	This work
pTHCas9noxD1	pTHCas9 with a 20 bp spacer targeting the *noxD* gene of *L*. *lactis* NZ9000; Erm^r^	This work
pTHCas9galk2	pTHCas9 with a 25 bp spacer targeting the *galK* gene of *L*. *lactis* NZ9000; Erm^r^	This work
pTHCas9hemN2	pTHCas9 with a 25 bp spacer targeting the *hemN* gene of *L*. *lactis* NZ9000; Erm^r^	This work
pTHCas9recA2	pTHCas9 with a 25 bp spacer targeting the *recA* gene of *L*. *lactis* NZ9000; Erm^r^	This work
pTHCas9noxD2	pTHCas9 with a 25 bp spacer targeting the *noxD* gene of *L*. *lactis* NZ9000; Erm^r^	This work
pTHCas9galk3	pTHCas9 with a 30 bp spacer targeting the *galK* gene of *L*. *lactis* NZ9000; Erm^r^	This work
pTHCas9hemN3	pTHCas9 with a 30 bp spacer targeting the *hemN* gene of *L*. *lactis* NZ9000; Erm^r^	This work
pTHCas9recA3	pTHCas9 with a 30 bp spacer targeting the *recA* gene of *L*. *lactis* NZ9000; Erm^r^	This work
pTHCas9noxD3	pTHCas9 with a 30 bp spacer targeting the *noxD* gene of *L*. *lactis* NZ9000; Erm^r^	This work
pTHCas9upp	pTHCas9 with a 25 bp spacer targeting the *upp* gene of *L*. *lactis* NZ9000; Erm^r^	This work
pTHCas9galk	pTHCas9 with a 25 bp spacer targeting the *galk* gene of *L*. *lactis* NZ9000; Erm^r^	This work
pL-beta	λ Red beta cloning in pLeiss:Sec:Nuc; Cm^r^	This work
pL-RecT	RecT from *E*. *faecalis* ATCC14506 cloning in pLeiss:Sec:Nuc; Cm^r^	This work
pL-LC50	LCABL_13050 from *Lb*. *casei* BL23 cloning in pLeiss:Sec:Nuc; Cm^r^	This work
pL-Lp41	Lp_0641 from *Lb*. *plantarum* WCFS1 cloning in pLeiss:Sec:Nuc; Cm^r^	This work
pL-JB02	phiJB_00020 from *Lb*. *delbrueckii* SDMCC050201 cloning in pLeiss:Sec:Nuc; Cm^r^	This work
pL-pi12	pi12 from *L*. *lactis* IL1403 cloning in pLeiss:Sec:Nuc; Cm^r^	This work

### Molecular manipulation

Bacteria genomic DNA extraction was carried out using a TIANamp Bacteria DNA kit (TIANGEN, China). Plasmids from *E*. *coli* were extracted using a Plasmid Mini Kit (Omega, USA). Restriction enzymes, T4 polynucleotide kinase and T4 DNA ligase were used as stated by standard procedures from New England Biolabs (NEB). High-fidelity DNA polymerase for cloning purposes and Taq polymerase for screening purposes were purchased from NEB and Takara (Japan), respectively. Primers and oligonucleotides were supplied by Tsingke Biological Technology Ltd. (Tsingke, China) and listed in Additional file 1: Table S1. DNA sequencing was performed by Biosune Biotechnology., Ltd. (Shanghai, China).

### Selection of functional single-stranded DNA binding protein for *L*. *lactis*

Six ssDNA proteins, Redβ of *E*. *coli* prophage λ, RecT of *E*. *faecalis* ATCC14506, LCABL_13050 of *Lb*. *casei* BL23, Lp_0641 of *Lb*. *plantarum* WCFS1, phiJB_00020 of *Lb*. *delbrueckii* SDMCC050201 and pi12 of *L*. *lactis* IL1403 were tested for the functionality and efficiency of ssDNA recombination in *L*. *lactis*. *Redβ* was amplified from pKD46 with primers RedβF and RedβR. *recT*, *LCABL_13050*, *Lp_0641*, *phiJB_00020* and *pi12* were amplified from the genomic DNA of strain ATCC14506, BL23, WCFS1, SDMCC050201 and IL1403 with primer pairs RecF/RecR, LC50F/LC50R, LP41F/LP41R, JB02F/JB02R and Pi12F/Pi12R, respectively. The yielding DNA fragments were digested and inserted into the compatible sites of the vector pSec:Leiss:Nuc, generating the recombinant plasmids pL-beta, pL-RecT, pL-LC50, pL-LP41, pL-JB02 and pL-pi12, respectively.

*Lactococcus lactis* NZ9000 carrying one of the above six recombinant plasmids was made electro-competent cells according to the previous method with some modification [42]. Briefly, cultures were grown at 30 °C in GM17 medium supplemented with 1% (wt/vol) glycine and 17% (wt/vol) sucrose. Nisin was added at an OD_600_ of 0.2 for induction of expression of the recombinase. Cells were harvested at an OD_600_ of 0.4, washed twice in ice-cold 10% glycerol supplemented with 17% (wt/vol) sucrose, and concentrated 125-fold. 100 μg (or 40, 20, 15, 10, 5, 1 μg) oligonucleotides were mixed with the competent cells and transferred to pre-chilled 0.2 cm electroporation cuvettes, and then electroporated at 2000 V, 20 μF and 200 Ω. Cells were recovered in 1 mL SGM17 medium (GM17 supplemented with 7.63% sucrose, 0.4% MgCl_2_, and 2.2% CaCl_2_) for 2 h at 30 °C and plated onto GM17 agar selective plates. To screen easily, the *rpoB* gene (locus tag llmg_1982) encoding DNA-directed RNA polymerase subunit β was employed as a target [19]. Oligonucleotide rpoBo1 was designed to introduce a H486 N point mutation, yielding a rifampicin-resistant phenotype. Viable cells on the GM17 agar plates containing rifampicin were detected by a mismatch amplification mutation assay-PCR (MAMA-PCR) with primers rpoB-MAMAF1, rpoB-MAMAF2 and rpoB-MAMAR [19].

### Construction of CRISPR/Cas9 vectors adapted to *L*. *lactis*

Plasmid pCas9 was purchased from Addgene (plasmid #42876). To adapt CRISPR/Cas9 to *L*. *lactis*, DNA fragment containing tracrRNA, cas9 and crRNA was PCR amplified from the plasmid pCas9 with primers 9-1F and 9-1R, and inserted into the BglII/PstI sites of pTRKL_2_ or pTRKH_2_, generating vector pTLCas9 or pTHCas9.

### New spacer adding into pTLCas9 and pTHCas9

To target the *upp* gene (locus tag llmg_2176) in the genome of *L*. *lactis* NZ9000, oligos upp-sp-20F/upp-sp-20R and upp-sp-25F/upp-sp-25R were phosphorylated and annealed. The products were diluted and inserted into the BsaI digested pTLCas9 and pTHCas9, generating pTLCas9upp and pTHCas9upp, respectively. To target the *galK* gene (locus tag llmg_2235), *hemN* gene (locus tag llmg_1418), *recA* gene (locus tag llmg_0374) and *noxD* gene (llmg_pseudo_74) in the genome of *L*. *lactis* NZ9000, oligos galk-sp-20F/galk-sp-20R, hemN-sp-20F/hemN-sp-20R, recA-sp-20F/recA-sp-20R and noxD-sp-20F/noxD-sp-20R were phosphorylated and annealed to produce spacers, respectively. The spacers were diluted and inserted into the digested pTLCas9 or pTHCas9, generating pTLCas9galK1, pTLCas9hemN1, pTLCas9recA1, pTLCas9noxD1, pTHCas9galK1, pTHCas9hemN1, pTHCas9recA1 and pTHCas9noxD1, respectively.

### Optimization of the length of spacer

To find the optimal length of spacer, the 25 bp and 30 bp spacers targeting the same position as the 20 bp spacer of the *galK* gene were also inserted into pTHCas9, generating pTHCas9galK2 and pTHCas9galK3. With the similar strategy, the 25 bp and 30 bp spacers targeting the *hemN*, *recA* and *noxD* gene were inserted into pTHCas9, respectively, generating pTHCas9hemN2, pTHCas9hemN3, pTHCas9recA2, pTHCas9recA3, pTHCas9noxD2 and pTHCas9noxD3.

### Gene point mutation, genomic DNA deletion and insertion

*Lactococcus lactis* NZ9000 recombinant carrying plasmid pL-RecT were made electro-competent cells as the above method. To describe the detail protocols, we took site-specific mutagenesis of *upp* gene as an example. The prepared competent cells were co-transformed with 10 μg of oligonucleotides uppo and 100 ng of target plasmid pTLCas9upp. After recovery, cells were plated on GM17 agar containing erythromycin. The genome of colonies containing desirable modification will not be cleaved by Cas9, and therefore the viable cells on the selective plate are potential recombinants. The candidates were tested by the growth in the GM9 medium supplemented with 5-fluorouracil and PCR amplification with primers upp-tF/upp-tR, and further confirmed by sequencing. The plasmid pTHCas9upp was cured by streaking the Δ*upp* mutant harboring pTHCas9upp on the GM17 agar plates without erythromycin. Then, the picked colonies that were grown on GM17 medium but not grown on GM17 medium containing erythromycin were considered as the pTHCas9upp free.

The other genomic engineering experiments were performed as the above protocol. For site-specific mutagenesis of the *galK* gene, the target plasmid pTHCas9galK2 and oligonucleotides galko1 were used. The mutants were tested by PCR amplification with primers gal-tF/gal-tR, and further confirmed by sequencing analysis. For genomic DNA deletion and insertion, the target plasmid pTHCas9noxD2 and oligonucleotides noxDo1 (50 bp deletion), noxDo2 (100 bp deletion) or noxD-loxPo (*loxP* site insertion) were used. The mutants were tested by PCR amplification with primers noxD-del-tF/noxD-del-tF, and confirmed by sequencing analysis.

### Statistical analysis

Statistical analysis was performed using unpaired two-tailed Student’s t tests. *P *< 0.05 were considered statistically significant. *P *< 0.01 were considered statistically high significant.

## Additional file

**Additional file 1: Figure S1.** Confirmation of *rpoB* H486N recombineered colonies. (A) MAMA-PCR based confirmation. (B) Sequence comparison between wild type and the designed mutant. (C) Sequence analysis of the colonies with rifampin resistance. In panel (B), mutations introduced by the ssDNA oligonucleotide are shown, with H486N missense mutation indicated as bold and bases as red. **Table S1.** Primers and oligonucleotides used in this study.

## Abbreviations

LAB: lactic acid bacteria
GRAS: generally regarded as safe
ssDNA: single-standed DNA
CRISPR: clustered regularly interspaced short palindromic repeat
DSB: double stranded break
PAM: protospacer-adjacent motif
MAMA-PCR: mismatch amplification mutation assay-PCR
