# Supplementary material for: A rapid and versatile tool for genomic engineering in Lactococcus lactis
Source: Microb Cell Fact. 2019 Jan 31;18:22. doi: 10.1186/s12934-019-1075-3 (PMC6357491; doi:10.1186/s12934-019-1075-3)
Supplement: Supplementary file 1 — Additional file 1: Figure S1. Confirmation of rpoB H486N recombineered colonies. (A) MAMA-PCR based confirmation. (B) Sequence comparison between wild type and the designed mutant. (C) Sequence analysis of the colonies with rifampin resistance. In panel (B), mutations introduced by the ssDNA oligonucleotide are shown, with H486N missense mutation indicated as bold and bases as red. Table S1. Primers and oligonucleotides used in this study. [file 12934_2019_1075_MOESM1_ESM.docx]

**A rapid and versatile tool for genomic engineering in *Lactococcus lactis***

Tingting Guo^a#^, Yongping Xin^a#^, Yi Zhang^a^, Xinyi Gu^a^, Jian Kong^a*^

^a^ State Key Laboratory of Microbial Technology, Shandong University, No. 72 Binhai Road, Qingdao 266237, P. R. China

Running title: Genomic engineering in *L*. *lactis*

*Corresponding author: Jian Kong

#These authors contributed equally to this work.

Mailing address: No. 72 Binhai Road, Qingdao 266237, P. R. China

E-mail: kongjian@sdu.edu.cn.

Tel: +86 532 58632433

Table S1 Primers and oligonucleotides used in this study

| Primers | Sequence^a^ | Description |
| --- | --- | --- |
| 9-1F | TGTAGATCTACGAAATCATCCTGTGGAGCTT | Cloning of DNA fragment containing tracrRAN, cas9 and crRNA from pCas9 |
| 9-1R | ATGCTGCAGATCACACTACTCTTCTTTTG |  |
| galK-sp-20F | AAACCAAAAGAATGCAAACTAAATG | synthesis of a 20 bp spacer targeting the *galK* gene of *L*. *lactis* NZ9000 |
| galK-sp-20R | AAAACATTTAGTTTGCATTCTTTTG |  |
| galK-sp-25F | AAACGCACTCAAAAGAATGCAAACTAAATG | synthesis of a 25 bp spacer targeting the *galK* gene of *L*. *lactis* NZ9000 |
| galK-sp-25R | AAAACATTTAGTTTGCATTCTTTTGAGTGC |  |
| galK-sp-30F | AAACGTGAAGCACTCAAAAGAATGCAAACTAAATG | synthesis of a 30 bp spacer targeting the *galK* gene of *L*. *lactis* NZ9000 |
| galK-sp-30R | AAAACATTTAGTTTGCATTCTTTTGAGTGCTTCAC |  |
| hemN-sp-20F | AAACAGCGAAGAATGATTAAATAGG | synthesis of a 20 bp spacer targeting the 5’ untranslated region of the *hemN* gene of *L*. *lactis* NZ9000 |
| hemN -sp-20R | AAAACCTATTTAATCATTCTTCGCT |  |
| hemN -sp-25F | AAACGGGATAGCGAAGAATGATTAAATAGG | synthesis of a 25 bp spacer targeting the 5’ untranslated region of the *hemN* gene of *L*. *lactis* NZ9000 |
| hemN -sp-25R | AAAACCTATTTAATCATTCTTCGCTATCCC |  |
| hemN -sp-30F | AAACGGAATGGGATAGCGAAGAATGATTAAATAGG | synthesis of a 30 bp spacer targeting the 5’ untranslated region of the *hemN* gene of *L*. *lactis* NZ9000 |
| hemN -sp-30R | AAAACCTATTTAATCATTCTTCGCTATCCCATTCC |  |
| recA-sp-20F | AAACGGTCGTATCGTTGAAATCTAG | synthesis of a 20 bp spacer targeting the *recA* gene of *L*. *lactis* NZ9000 |
| recA-sp-20R | AAAACTAGATTTCAACGATACGACC |  |
| recA-sp-25F | AAACCTAAAGGTCGTATCGTTGAAATCTAG | synthesis of a 25 bp spacer targeting the *recA* gene of *L*. *lactis* NZ9000 |
| recA-sp-25R | AAAACTAGATTTCAACGATACGACCTTTAG |  |
| recA-sp-30F | AAACTTACCCTAAAGGTCGTATCGTTGAAATCTAG | synthesis of a 30 bp spacer targeting the *recA* gene of *L*. *lactis* NZ9000 |
| recA-sp-30R | AAAACTAGATTTCAACGATACGACCTTTAGGGTAA |  |
| noxD-sp-20F | AAACGATAAATTAATTTTAGCGACG | synthesis of a 20 bp spacer targeting the *noxD* gene of *L*. *lactis* NZ9000 |
| noxD-sp-20R | AAAACGTCGCTAAAATTAATTTATC |  |
| noxD-sp-25F | AAACATTATGATAAATTAATTTTAGCGACG | synthesis of a 25 bp spacer targeting the *noxD* gene of *L*. *lactis* NZ9000 |
| noxD-sp-25R | AAAACGTCGCTAAAATTAATTTATCATAAT |  |
| noxD-sp-30F | AAACTACCGATTATGATAAATTAATTTTAGCGACG | synthesis of a 30 bp spacer targeting the *noxD* gene of *L*. *lactis* NZ9000 |
| noxD-sp-30R | AAAACGTCGCTAAAATTAATTTATCATAATCGGTA |  |
| upp-sp-20F | AAACCGTGAGCTTGTTGACGAAATG | synthesis of a 20 bp spacer targeting the *upp* gene in *L*. *lactis* NZ9000 |
| upp-sp-20R | AAAACATTTCGTCAACAAGCTCACG |  |
| upp- sp-25F | AAACAATTCCGTGAGCTTGTTGACGAAATG | synthesis of a 25 bp spacer targeting the *upp* gene in *L*. *lactis* NZ9000 |
| upp- sp-25R | AAAACATTTCGTCAACAAGCTCACGGAATT |  |
| gal-spF | AAACTTCCTCGTCTTGAACTGGTTCAATTG | synthesis of spacer targeting the *galK* gene to be mutated in *L*. *lactis* NZ9000 |
| gal-spR | AAAACAATTGAACCAGTTCAAGACGAGGAA |  |
| RedαβF | TAGATATCATGAGTACTGCACTCGCAACGCT | Cloning of λ Red α and β from plasmid pKD46 |
| RedαβR | GAGAAGCTTTCATCGCCATTGCTCCCCA |  |
| RecF | AACTGCAGAGATGAGTAACGATTTAACACAAATGA | Cloning of RecT from *E*. *faecalis* ATCC14506 |
| RecR | ACAGGTCGACTCAGAAAGGATAATCGTCTTCTTC |  |
| LC50F | AACTGCAGATGACGACACAATATGACCTAAA | Cloning of LCABL_13050 from *Lb*. *casei* BL23 |
| LC50R | ACAGGTCGACTTATCCTTGTTGACCGTCAA |  |
| LP41F | AACTGCAGATGAGTAATGAGCTAGTTACGATGG | Cloning of Lp_0641 from *Lb*. *plantarum* WCFS1 |
| LP41R | ATACTCGAGTTAGCTGGCGTCAAAGTCTC |  |
| JB02F | AACTGCAGATGTCAATGTTAGATGTACT | Cloning of phiJB_00020 from *Lb*. *delbrueckii* SDMCC050201 |
| JB02R | ACAGGTCGACCTAGAATGGCAGGTCGTCTT |  |
| Pi12F | AACTGCAGAGATGAATAAAAGATTAAGTT | Cloning of pi12 from *L*. *lactis* IL1403 |
| Pi12R | ACAGGTCGACTTAAATTAAATCTGAATACGAA |  |
| rpoB-MAMAF1 | TGTGACTCGTGATGTGCTCGATTTA | MAMA-PCR amplification of the mutant *rpoB* gene |
| rpoB-MAMAF2 | CATAATCCTTTGTCTGAGCTTAGCA |  |
| rpoB-MAMAR | ACGGTTGGAGTCATCGTTTTC |  |
| gal-tF | TTGACTTAGATGATGTTGA | PCR amplification of the mutant *galK* gene |
| gal-tR | ACTTCGTAATCATCTTTTA |  |
| noxD-del-tF | GCAGATGGAAACTGTGATAGAAG | PCR amplification of the mutant *noxD* gene |
| noxD-del-tR | TAGCTGTTGCCAAAAATTCTTC |  |
| upp-tF | ATGTCAAAATTTCAAGTCGTA | PCR amplification of the mutant *upp* gene |
| upp-tR | TTCATTAAGTTTTTCATCCAA |  |
| Oligonucleotides |  |  |
| rpoBo1 | GAGATACCACCAGGTCCTAAGGCAGAGAAACGACGTTTGTTGCTAAGCTCAGACAAAGGATTATGTTGGTCCATAAATTGT | ssDNA for site-specific mutagenesis of the *rpoB* gene |
| galKo | GGAGTTGACACCAATATAATCATTTTCTGTTTTTTGTTTTGGTTGAACCAGTTCAAGACGAGGAACACTTAGCTTAAATA | ssDNA for site-specific mutagenesis of the *galK* gene |
| uppo | AGGTAAATCTCTTGATACTTCGTAGGCCATAAGCATTTATTATTCGTCAACAAGCTCACGGAATTCTTTTGTTGACGCCT | ssDNA for site-specific mutagenesis of the *upp* gene |
| noxDo1 | AGATTACTTTTTTTATCAAAAGGAACTGAGGTCATCGCCGTTCTTTGAGTTACTTCTACGGTAAAATACTTTTTTATGAT | ssDNA for 50-bp deletion of the *noxD* gene |
| noxDo2 | CCAAAAATTCTTCAGCCTTTATTGCATCTTCCATACTCTTTTCTTTGAGTTACTTCTACGGTAAAATACTTTTTTATGAT | ssDNA for 100-bp deletion of the *noxD* gene |
| noxD-loxPo | AAGGAACTGAGGTCATCGCCGGATATGAGCCCATAACTTCGTATAGCATACATTATACGAAGTTATACTTTTGTTTTCTTTGAGTTACTTCTACGGTAA | ssDNA for 34-bp loxP site insertion into the *noxD* gene |

^a^Restriction sites for ligation are underlined.


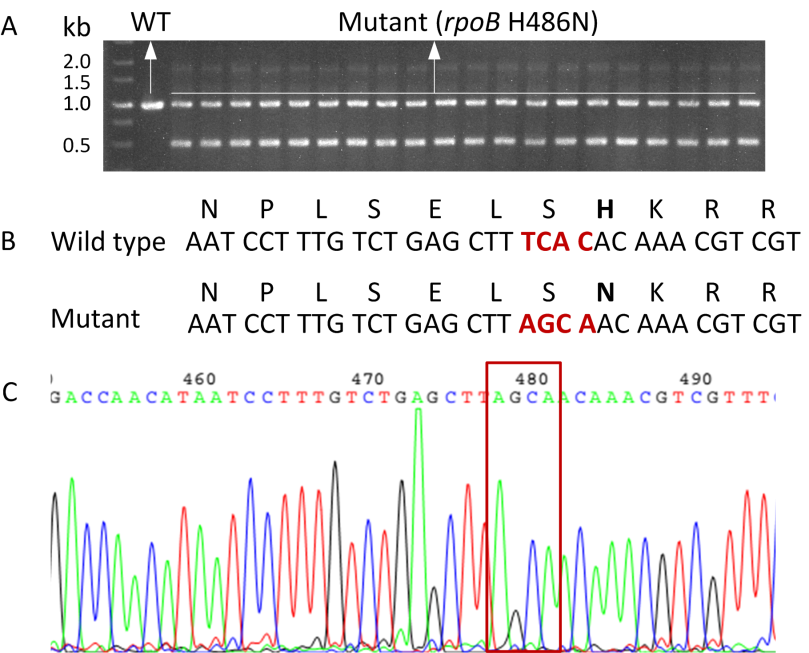


Figure. S1. Confirmation of *rpoB* H486N recombineered colonies. (A) MAMA-PCR based confirmation. (B) Sequence comparison between wild type and the designed mutant. (C) Sequence analysis of the colonies with rifampin resistance. In panel (B), mutations introduced by the ssDNA oligonucleotide are shown, with H486N missense mutation indicated as bold and bases as red.
